# Surgical Treatment of Wilkie’s Syndrome by Vascular Transposition

**DOI:** 10.7759/cureus.24251

**Published:** 2022-04-18

**Authors:** Talal Ali, Jan Tomka, Ilkin Bakirli, Ifrat Bakirov

**Affiliations:** 1 Vascular Surgery Department, National Institute of Cardiovascular Diseases and Slovak Medical University, Bratislava, SVK; 2 General Surgery Department, Imam Abdulrahman Alfaisal Hospital, Riyadh, SAU

**Keywords:** abdominal aorta, duodenal obstruction, superior mesenteric artery, dunbar syndrome, renal nutcracker syndrome, ligament of treitz, duodenojejunostomy, infrarenal transposition of the superior mesenteric artery, superior mesenteric artery syndrome, wilkie’s syndrome

## Abstract

Introduction

Superior mesenteric artery syndrome (SMAS), also called mesenteric duodenal compression syndrome, Wilkieʼs syndrome, chronic duodenal ileus or cast syndrome, is a rare clinical condition defined as a compression of the third portion of the duodenum in between the SMA and abdominal aorta (AA), due to narrowing of the space between them. SMAS is primarily attributed to loss of the intervening mesenteric fat pad, leading to partial or complete duodenal obstruction. Its manifestations are complex and non-specific, including postprandial epigastric pain, nausea, vomiting, early satiety, weight loss and anorexia. SMAS may present as an acute syndrome, or it may have an insidious onset with chronic symptoms. SMAS mainly affects females between 10 and 40 years of age. This study aims to discuss the safety and efficacy of vascular decompression of the duodenum by infrarenal transposition of SMA.

Methods

This single-centre prospective clinical study analysed 37 patients with Wilkie’s syndrome who underwent infrarenal transposition of the SMA between January 2012 and December 2021. The indications for the surgery were severe weight loss, uncontrolled upper abdominal pain, vomiting and other gastrointestinal (GI) symptoms that were severely debilitating to patients’ daily lives, along with radiological findings such as aortomesenteric angle < 25°, aortomesenteric distance <8 mm and distention of proximal part of the duodenum and the stomach. Ten patients (27%) concurrently had Nutcracker syndrome and seven patients (18.9%) had Dunbar syndrome (median arcuate ligament syndrome). Three female patients (8.1%) had all three above-mentioned vascular compression syndromes, which were treated in the same surgery. One male patient (2.7%) was after a laparoscopic duodenojejunostomy with symptoms that relapsed three months postoperatively, which was cured after the infrarenal transposition of SMA.

Results

Technical operative and clinical success were achieved in all patients. There were no cases of anastomotic failure, SMA thrombosis or intestinal ischemia. All of the patients are currently living symptom-free. One patient (2.7%), four days postoperatively, had a lymphocele formed in the retroperitoneum, which was successfully drained by a CT-guided percutaneous pigtail catheter. Another patient (2.7%) after three months of surgery needed a re-laparotomy for adhesive obstruction of the second part of the duodenum and was treated by adhesiolysis and omentoplasty. One patient (2.7%), 2-year postoperatively, had a proximal SMA stenosis up to 60% where drug-eluting balloon percutaneous transluminal angioplasty (DEB PTA) was performed successfully. Finally, the upper GI symptoms were resolved in all 37 patients (100%).

Conclusion

Wilkie’s syndrome, although rare, is frequently late-diagnosed or underdiagnosed. In cases of failure of conservative therapy, infrarenal transposition of the SMA can be considered a safe and feasible surgical option with more physiologically favourable outcomes comparable to gastrointestinal bypasses, especially in patients concurrently suffering from Nutcracker syndrome. Simultaneously, it also restores physiologic duodenal passage of gastroduodenal content without the need of creating a digestive tract anastomosis. To our best knowledge, we have the highest number of SMA transposition surgeries performed in a single centre for the treatment of Wilkie’s syndrome.

## Introduction

Superior mesenteric artery syndrome (SMAS), also called mesenteric duodenal compression syndrome, Wilkieʼs syndrome, chronic duodenal ileus or cast syndrome, is a rare type of small bowel obstruction that occur as a result of extrinsic compression of the third part of the duodenum in between the SMA and abdominal aorta (AA), due to narrowing of the space between them [1-3]. The Austrian professor Carl Freiherr von Rokitansky in 1842 described this condition as an internal hernia on the autopsy finding [4]. SMAS is primarily attributed to loss of the intervening mesenteric fat pad, leading to partial or complete duodenal obstruction. Multiple factors are associated with SMAS, mainly marked weight loss as a consequence of other diseases (cancer, bariatric surgery, chronic infections and severe burns) but may also be congenital in conditions such as short ligament of Treitz or abnormal origin of the SMA, or associated with surgical interventions that distort the anatomy as scoliosis correction surgery [1] or esophagectomy. Its manifestations are complex and non-specific, including postprandial epigastric pain (59%), nausea (40%), vomiting (50%), early satiety (32%), weight loss and anorexia (32%) [5]. SMAS may present as an acute syndrome, or it may have an insidious onset with chronic symptoms. SMAS has an estimated prevalence among the general population that varies between 0.013 and 0.3% [6], and it mainly affects females between 10 and 40 years of age. This study aims to discuss the safety and efficacy of vascular decompression of the duodenum by infrarenal transposition of SMA in Wilkieʼs syndrome.

Duodenum is the initial part of the small bowel and has four parts. The third portion of the duodenum passes from right to left between the AA and the SMA at the third lumbar vertebral body level. The last part of the duodenum is suspended by its attachment to the ligament of Treitz. The SMA is a visceral branch of AA, that arises from the anterior aspect of the aorta just below the celiac trunk at the level of the L1 vertebral body and crosses the third portion of the duodenum anteriorly. It is enveloped with connective tissue elements, like fatty tissue and lymphatic tissues, and extends downwards at an acute angle into the mesentery. In the majority of healthy individuals, the normal angle between the SMA and the AA is between 30º and 45º and the aortomesenteric distance is 10 to 28 mm [7,8], due in part to the mesenteric fat pad. However, in Wilkie’s syndrome, the aortomesenteric angle is narrowed to <25° and the distance between AA and SMA is shortened to < 8 mm.

## Materials and methods

A total of 37 patients with Wilkie’s syndrome were enrolled in this study to undergo the SMA transposition surgery. The approval for the study was taken from Institutional Review Board of National Institute of Cardiovascular Diseases (Approval Number: 24.02.22). Patients were treated at the Department of Vascular Surgery in the National Institute for Cardiovascular Diseases in Bratislava, Slovakia, for a period of 10 years (from January 2012 to December 2021). The following factors were analysed: demographic data, physiological factors, laboratory factors, imaging study characteristics and time between the onset of symptoms and the surgery, as well as patients’ follow-up. All the factors were correlated with the in-hospital database. Patient characteristics were as follows 28 female (75.6%) and nine male (24.4%), with mean ages of 34.5 (14-62) and 27 (19-29), respectively (Table 1).

**Table 1:** Gender distribution and age ranges of patients.

Gender	Occurrence	Percentage	Age range
Male	9	24.4%	19-29
Female	28	75.6%	14-62
Total	37	100%	14-62

Ten patients (27%), eight females and two males, concurrently had Nutcracker syndrome and seven patients (18.9%), seven females and three males had Dunbar syndrome (median arcuate ligament syndrome). Three female patients (8.1%) had all three above-mentioned vascular compression syndromes (Table 2).

**Table 2:** Occurrence and combination of vascular compression syndromes. W: Wilkie’s syndrome; N: Nutcracker syndrome; D: Dunbar syndrome

Syndrome	Male	Female	Total	Percentage
W	4	13	17	46%
W + N	2	8	10	27%
W + D	4	3	7	18.9%
W + N + D		3	3	8.1%

Dunbar syndrome was treated in the same surgery by dividing the median arcuate ligament, ligating the celiac ganglion and performing adhesiolysis of the fibrous tissues around the celiac trunk. Infrarenal transposition of SMA simultaneously releases the compression of the left renal vein (LRV), so no additional action is required for the management of concurrently existing Nutcracker syndrome. One male patient (2.7%) was after a laparoscopic duodenojejunostomy, with symptoms that relapsed three months after the surgery.

**Figure 1:**
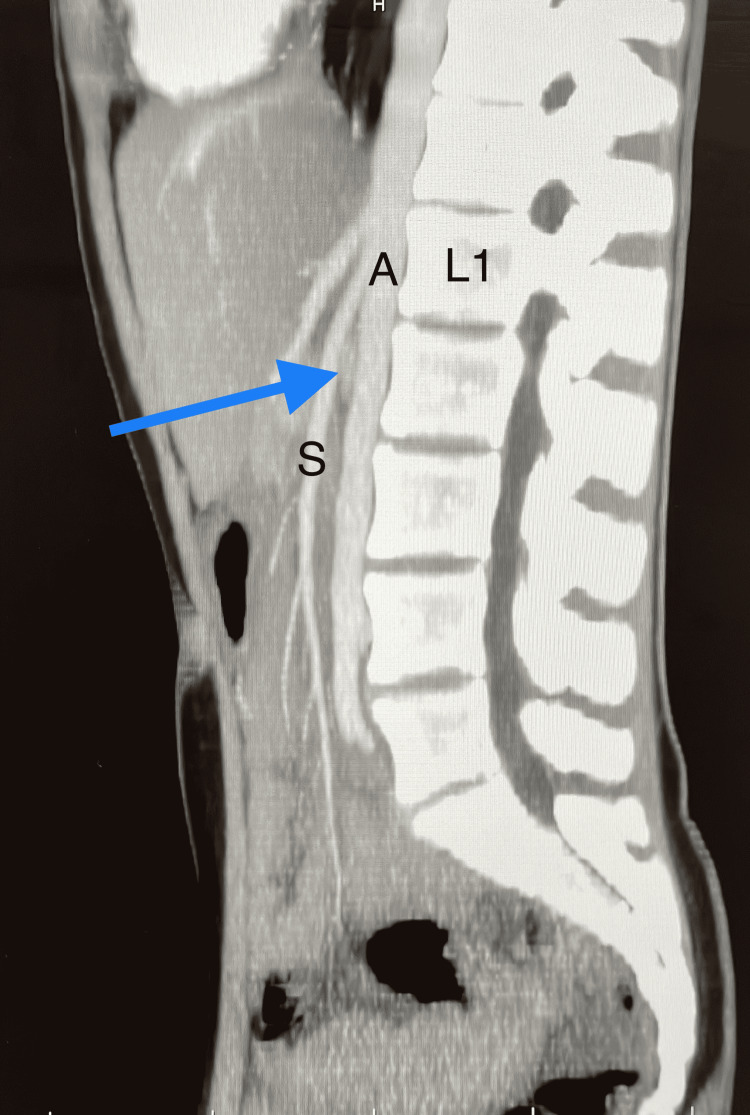
Arterial phase sagittal view of the aortomesenteric angle of 14 degrees. L1: 1st lumbar vertebra; S: superior mesenteric artery; A: abdominal aorta; Blue arrow: compressed left renal vein

All the included patients had undergone a complete gastroenterological examination, including upper GI endoscopy, barium contrast series and computed tomography angiography (CTA), to exclude any other cause of duodenal obstruction and to confirm the diagnosis of SMAS. In four patients, in addition to the studies mentioned above, magnetic resonance angiography (MRA) imaging was used. Ultrasonography (USG) was mainly utilised to exclude biliary pathologies. CTA and MRA enable visualisation of the vascular compression of the duodenum and precise measurement of aortomesenteric angle and distance [9]. CTA was the most reliable imaging modality to diagnose SMAS, as also mentioned by Lee et al. [10]. Early diagnosis is crucial, as delays in diagnosis can result in a chronic course of symptoms, with marked disturbances in fluid and electrolyte balances. The diagnostic features of imaging modalities included (a) narrowing of the aortomesenteric angle to <25° (Figure 1); (b) shortening of the distance between the SMA and AA to <8 mm; (c) distension of the stomach and proximal part of the duodenum; (d) and in some cases compression and distention of the LRV.

The patients selected were those who had failed conservative therapy and presented with chronic or acute chronic symptoms, such as those mentioned before, which were severely debilitating to the patients’ daily lives. Exclusion criteria were as follows: (a) patients with radiological findings suggestive of Wilkie’s syndrome but without symptomology (2 patients); (b) anatomical variations such as celiacomesenteric trunk or common hepatic artery being a branch of the SMA, which potentially could cause technical difficulties and possible complications during or after the surgery (1 patient ).

Current therapy of SMAS consists of medical and/or surgical treatment. Conservative therapy includes nutritional support, left-lateral decubitus positioning, prokinetic and anti-reflux medications and fluid resuscitation [6]. It is the conviction of most authors that a trial of medical treatment should be attempted before any surgical intervention [11]. However, there is neither clear time limit for the long-term outcome of conservative therapy. Failure of conservative medical therapy or recurrent episodes of the symptoms are indications for surgical treatment.

Surgical approaches include Strong’s procedure (a division of the ligament of Treitz) and GI bypasses such as open or laparoscopic duodenojejunostomy, gastrojejunostomy, or Roux-en-Y duodenojejunostomy. Traditionally open duodenojejunostomy, and recently laparoscopic duodenojejunostomy, are considered to be the operation of choice to relieve the obstruction with a high success rate [11-13]. Nonetheless, it has several postoperative complications related to intervening within GI tract continuity. Yilnen et al. have reported a 7-year follow-up study of 16 patients treated with duodenojejunostomy and found that outcome was regarded as excellent by three patients, good by six, satisfactory by five, and poor by two patients [13]. Complications of GI bypasses include anastomotic or staple line leak, postoperative haemorrhage, bowel obstruction, incorrect Roux limb reconstruction and dumping syndrome causing diarrhoea, nausea or vomiting. Long-term complications may be challenging to differentiate from other GI disorders and include anastomotic stricture, marginal ulceration and perforation, fistula formation and nutritional deficiencies.

The first infrarenal transposition of the SMA as a treatment for SMAS was described by Pourhassan et al. in 2008 in Dusseldorf University Hospital, Germany [14]. Since then, many single or small group case reports of performing this surgery have been published. It is the standard well-known technique of infrarenal transposition of SMA. Our technique is slightly different from this standard description. We prefer to make anastomosis at the right anterolateral aspect of the infrarenal aorta, for the best alignment of the mesentery. In addition to that, we are ligating the encountered branches of cisterna chyli to avoid postoperative lymph leakage and subsequent morbidities. After completing the anastomosis we are confirming the normal blood flow by intraoperative Doppler study.

**Figure 2:**
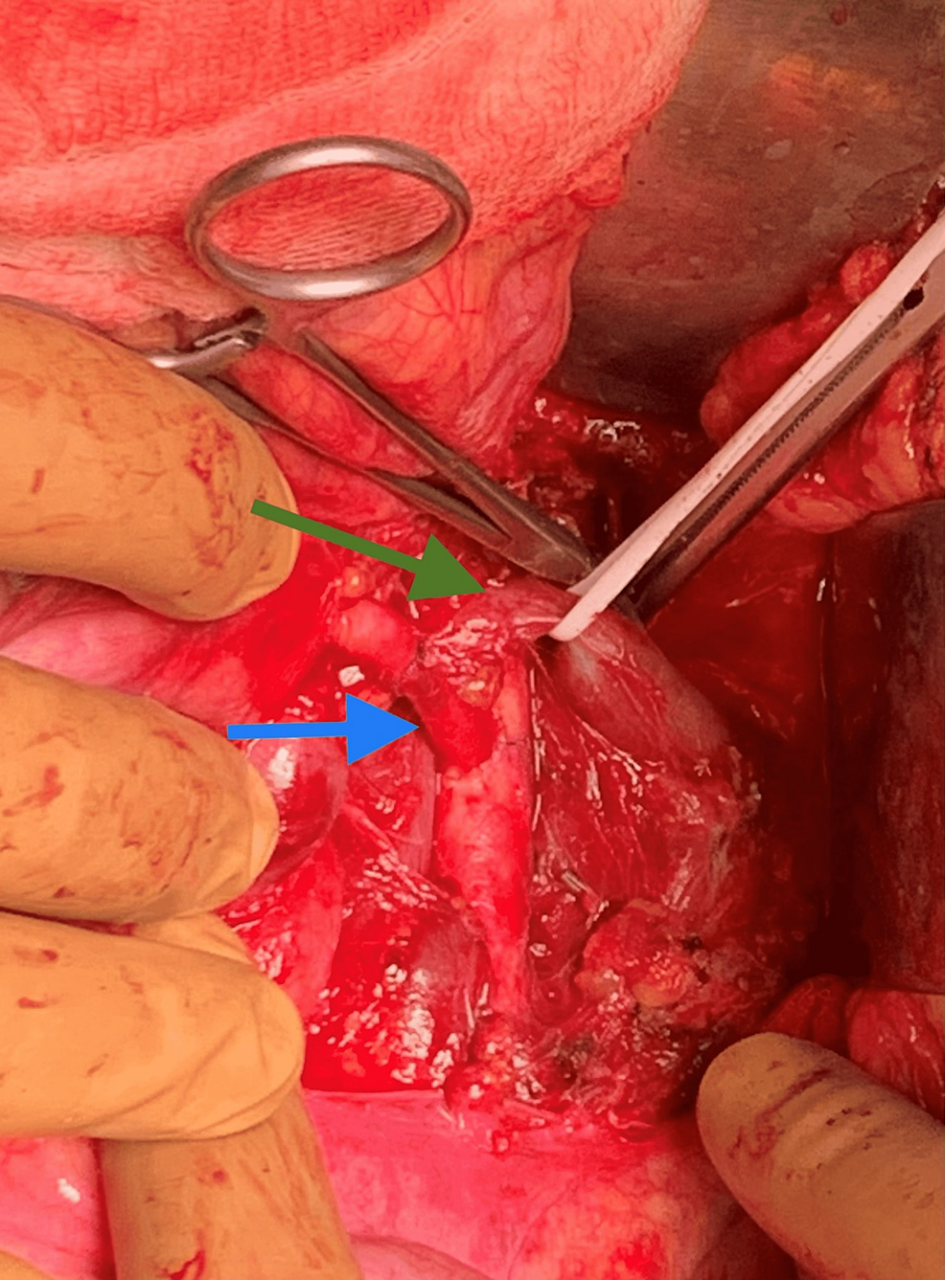
Infrarenal transposition of the superior mesenteric artery (blue arrow). Left renal vein (green arrow).

All our SMA transposition surgeries were performed under general anaesthesia, where an upper midline laparotomy is made (large incisions are not required, as all patients with Wilkie’s syndrome are underweight). The greater omentum and small bowel loops are packed outside the abdomen. The ligament of Treitz is divided to better mobilise the duodenum. Then the juxtarenal aorta is prepared in the retroperitoneum. All encountered branches of cisterna chyli are ligated to avoid unnecessary lymph leakage due to iatrogenic injury. The patient is heparinised, and the SMA is clamped proximally and distally and is cut at its proximal portion. The orifice is sutured and ligated with Prolene 5-0 suture. Then the aorta is partially clamped (tangentially) at the infrarenal level with a Satinsky clamp. A new orifice of 1 cm is created on the infrarenal part of the aorta on its right anterolateral aspect by the aortic punch, and the SMA is anastomosed end-to-side fashion with Prolene 5-0 in a single-stitch technique (Figure 2).

The clamps are released, deairing achieved and blood flow is checked by intraoperative Doppler. SMA clamping time is kept to under 5 minutes to avoid any risk of intestinal ischemia. After achieving perfect haemostasis, the wound is closed in layers. We prefer to suture the skin intradermally to minimise the visibility of the scar as most of the patients are young females. The patients are extubated after the surgery and transferred to the ward without the need for an intensive care unit. Postoperatively, the patient is left nil per os (NPO) for a day, then the patient is realimented starting with a liquid diet for the first day. The patients are usually discharged on the 3rd-4th postoperative day on a 6-month prescription of aspirin 100 mg once daily.

## Results

**Figure 3:**
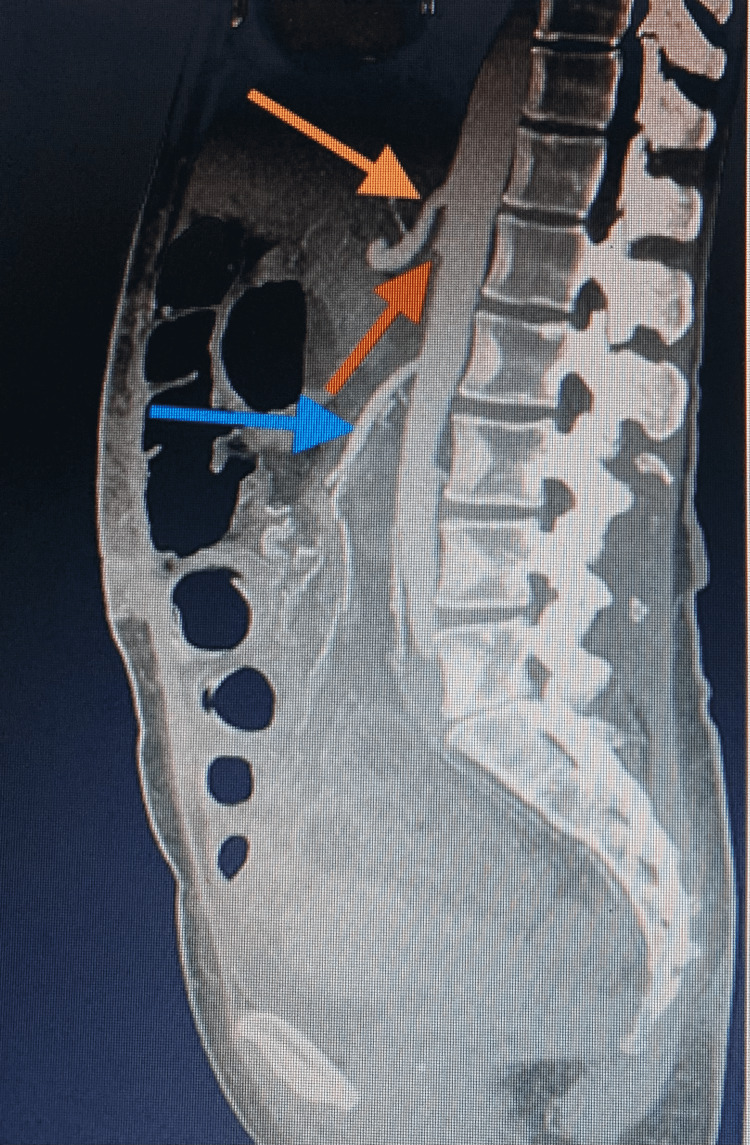
Control CTA scan. The superior mesenteric artery (blue arrow) after transposition with a normal aortomesenteric angle. Previous orifice of the superior mesenteric artery (red arrow) and celiac trunk (yellow arrow). CTA: computed tomography angiography

Patients were followed up in the first month after the surgery, then at 3, 6, 12, 24 and 48 months. In the early postoperative period, accelerated GI passage was observed in 12 patients (32.43%), with patients having more frequent bowel movements, which resolved in 2-3 weeks. The average weight gain in the first six months and one year were about 2.7 kg and 3.8 kg, respectively. Technical operative and clinical success were achieved in all patients. There were no cases of anastomotic failure, SMA thrombosis or intestinal ischemia. All of the patients are currently living symptom-free. Control CTA was done after three months from the day of surgery in the initial 10 operated patients as surveillance and found normal aortomesenteric angle in all 10 patients (Figure 3).

**Figure 4:**
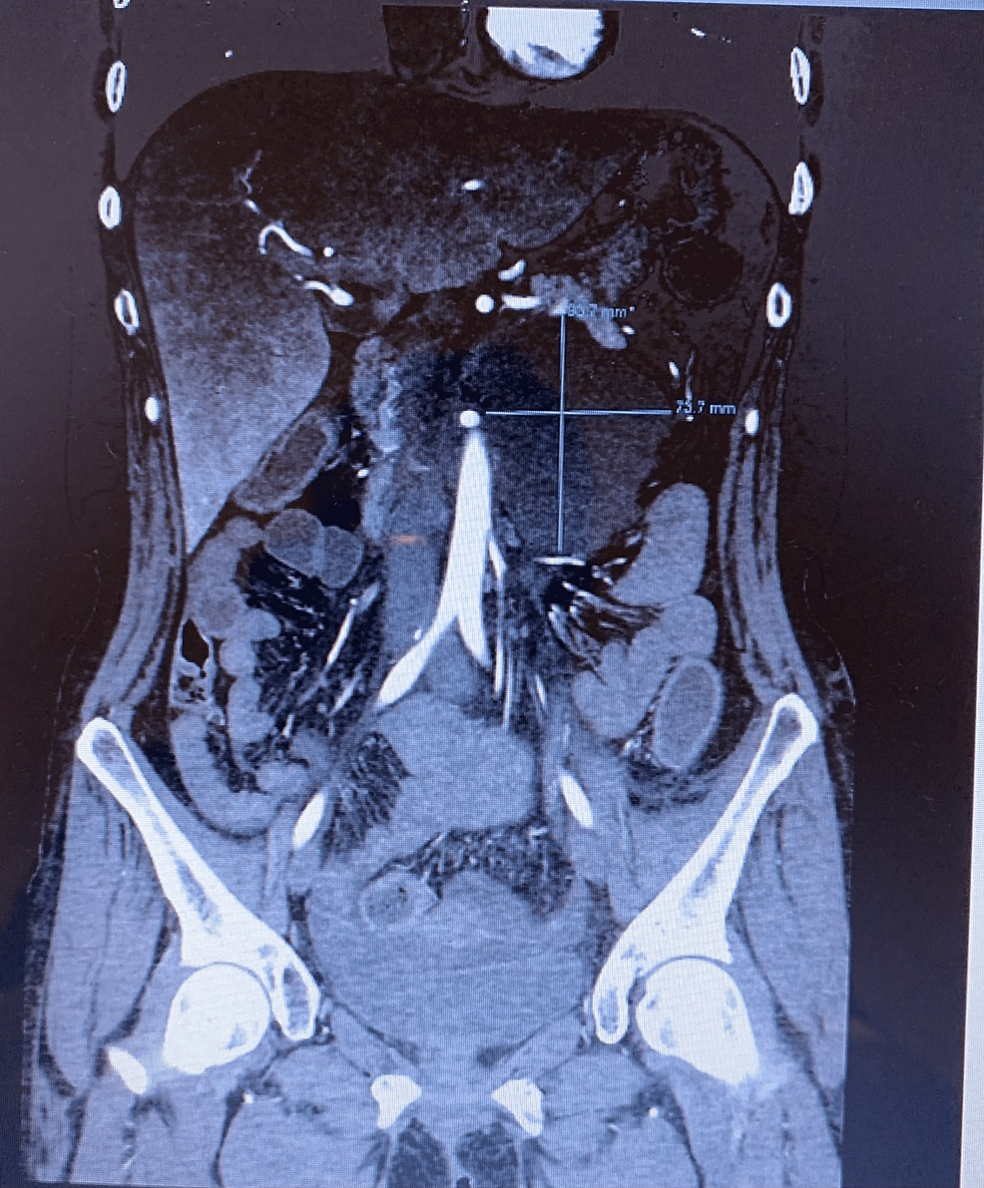
Retroperitoneal lymphocele.

**Figure 5:**
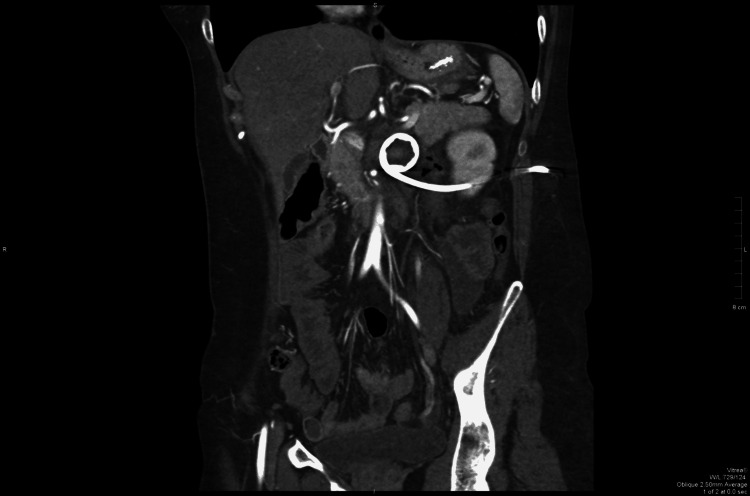
Percutaneous pigtail catheter drainage of lymphocele.

One patient (2.7%), four-day postoperatively, had a lymphocele formed in the retroperitoneum, which was successfully drained by a CT-guided percutaneous pigtail catheter (Figures 4-5).

**Figure 6:**
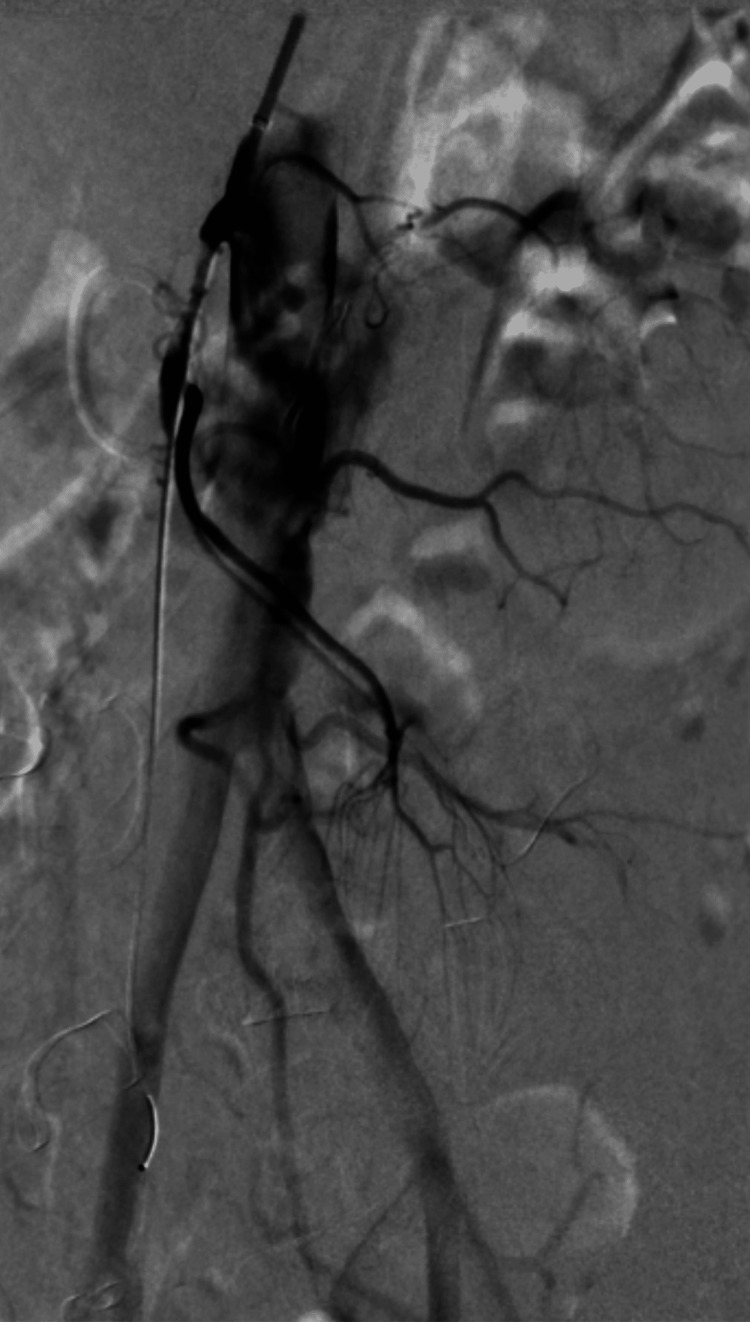
Selective angiography just before balloon dilatation of the superior mesenteric artery.

**Figure 7:**
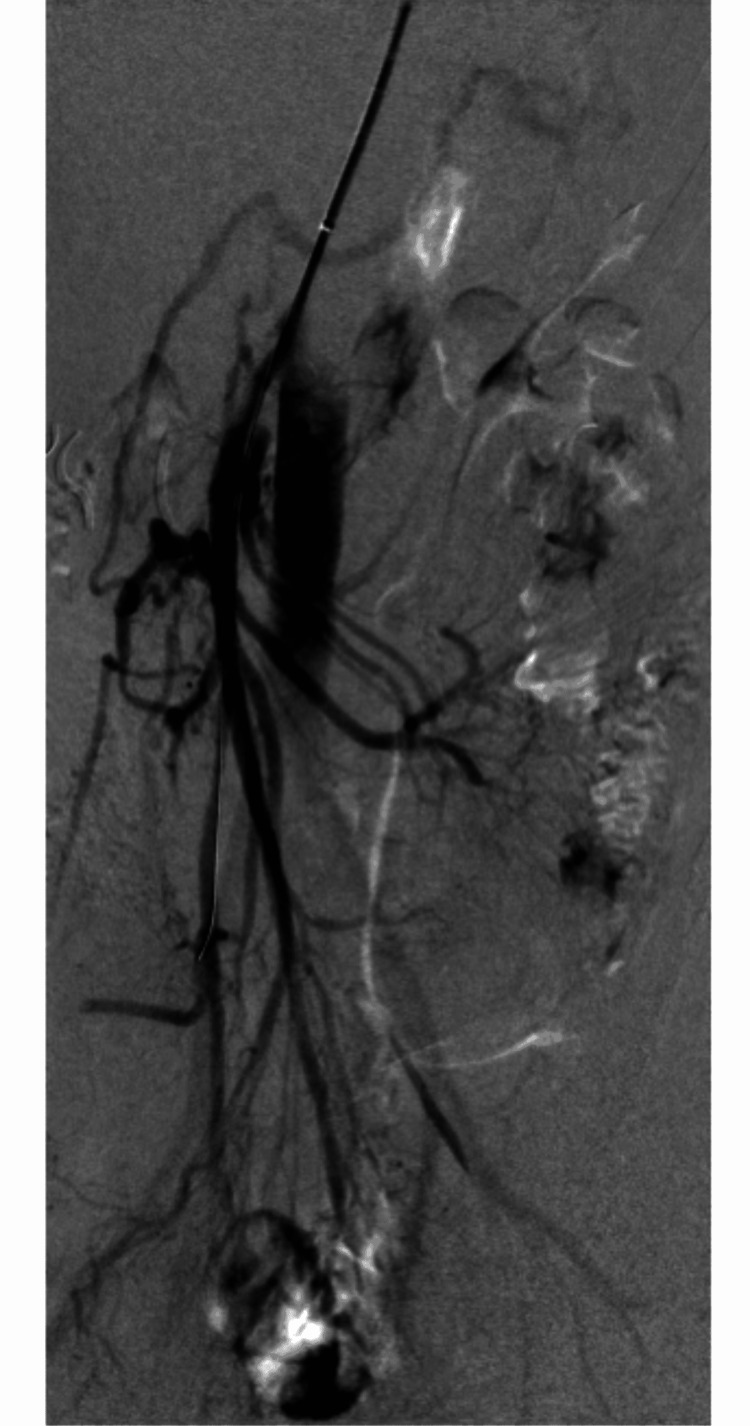
Balloon dilatation of the superior mesenteric artery.

One patient (2.7%) needed a relaparotomy 3-month postoperatively due to adhesions that had formed around the duodenum leading to duodenal compression in the second segment for which we performed adhesiolysis and omentoplasty. One patient (2.7%), 2-year postoperatively, had a proximal SMA stenosis up to 60% where drug-eluting balloon percutaneous transluminal angioplasty (DEB PTA) was performed successfully (Figures 6-7).

In this study, over the course of 10 years, we have performed 37 SMA transposition surgeries successfully with outstanding outcomes distinguished within regularly following up of our patients. Our study showed that the patients’ health had improved vastly after the surgery, both psychologically and physically, without the persistence of symptoms and without major complications. The average weight gain in a 6-month and one year was about 2.7 kg and 3.8 kg, respectively. All our patients were operated on by our most senior author (JT); it is highly recommended that the operators have advanced expertise in the anatomy of visceral arteries to provide the best possible outcome. Nutcracker syndrome/LRV entrapment syndrome. It refers to compression of the LRV, most commonly between AA and SM. The symptoms vary from asymptomatic haematuria, orthostatic proteinuria, flank pain, varicocele, dyspareunia and dysmenorrhea to severe pelvic congestion [15]. All 10 patients who had concomitantly SMAS and Nutcracker syndrome after vascular transposition have successfully recovered from both compressive syndromes, with no residual symptoms and signs.

## Discussion

Currently, open and laparoscopic duodenojejunostomy is considered the operation of choice in Wilkie’s syndrome, with a high success rate [11-13]. Nonetheless, it has several postoperative complications related to intervening with the GI tract continuity, like an anastomotic leak, intraperitoneal abscess formation, dumping syndrome, stenosis, ulceration at anastomosis or at the efferent limb. Performing the SMA transposition surgery not only preserves the continuity of the GI tract but also definitively relieves the compression of the 3rd part of the duodenum, along with other structures such as the LRV and the splanchnic nerves that innervate the duodenum. We believe that the reverse peristalsis can be the reason for residual symptoms after GI bypasses, which we did not observe in our patients after the SMA transposition surgery. It indirectly supports our idea that reverse peristalsis in Wilkie’s syndrome is possibly due to compression of the duodenal wall with its splanchnic nerves and also compression of the root of small bowel mesentery against the 3rd part of the duodenum. Another advantage of SMA transposition over GI anastomosis is that the patient undergoes a clean surgery instead of clean-contaminated, which minimises the infective complications like intraabdominal sepsis or wound infection.

Along with the surgical treatment of SMAS, psychological support should not be underestimated. Due to severe abdominal pain and difficulty in diagnosis of this syndrome, patients usually go undiagnosed for a prolonged period, which can be psychologically devastating to some of these patients. Some of Wilkie’s syndrome patients (6 in our study, 16.2%) also suffer from anorexia nervosa, which makes diagnoses even more complicated. Multidisciplinary teams, including gastroenterologists, general surgeons, vascular surgeons, and psychiatrists are necessary to diagnose and optimally treat this rare but potentially life-threatening syndrome.

This study has the following limitations (a) a limited number of patients, which is directly related to the rarity of Wilkie’s syndrome, within a 10-year period we could recruit only 40 patients and three of them were excluded from the study as per exclusion criteria; (b) our patients are not homogenously unified, in 20 patients SMAS coexisted with other intraabdominal vascular compression syndromes.

## Conclusions

Wilkie’s syndrome, although rare, is frequently late-diagnosed or underdiagnosed. In cases of failure of conservative therapy, infrarenal transposition of the SMA can be considered a safe and feasible surgical option with more physiologically favourable outcomes comparable to GI bypasses, especially in patients concurrently suffering from Nutcracker syndrome. Infrarenal transposition of the SMA achieves an etiological cure of Wilkie’s syndrome by releasing the compression of the duodenum, which in our belief is the possible cause of reverse peristalsis in such patients. Simultaneously, it also restores physiologic duodenal passage of gastroduodenal content without the need of creating a digestive tract anastomosis. We strongly recommend that transposition of SMA should be performed only by a well experienced vascular surgeon in a higher vascular centre, where angiography, interventional radiology and endovascular procedures are routine practice.

To our best knowledge, we have the highest number of SMA transposition surgeries performed in a single centre for the treatment of Wilkie’s syndrome.
